# Peer Review of “COVID-19 Infection and Symptoms Among Emergency Medicine Residents and Fellows in an Urban Academic Hospital Setting: Cross-sectional Questionnaire Study”

**DOI:** 10.2196/36207

**Published:** 2022-01-27

**Authors:** Reza Shahriarirad

**Affiliations:** 1 Shiraz University of Medical Sciences Shiraz Iran

**Keywords:** COVID-19, emergency medicine, housestaff wellness, medical education, training, frontline health care workers, frontline, personal protective equipment, pandemic, infectious disease, emergency

*This is a peer-review report submitted for the paper “COVID-19 Infection and Symptoms Among Emergency Medicine Residents and Fellows in an Urban Academic Hospital Setting: Cross-sectional Questionnaire Study.”*

## Round 1

1. The authors [1] describe a cross-sectional study on COVID-19 infection and symptom severity among emergency medicine residents and fellows in urban academic hospital settings. Their study consists of a rather small sample size of health care workers during the early period of the pandemic.

2. The authors noted a “high percentage of survey participation from the cohort” as a strength of their study, although only a 62% response rate was achieved, which is merely above the acceptable rate.

3. Abbreviations should be mentioned in full the first time they appear in the text, such as COVID-19, SARS, etc.

4. The manuscript should be edited for punctuation and grammatical errors.

5. Why wasn’t multiple regression or linear regression analysis performed?

6. I recommend reporting local countries’ protocols during the timeline of your study so that international readers could have insight into the situation and the protective measures applied during the study period.

7. SARS-CoV-2 is the virus that causes the infection, and COVID-19 is the disease. Proper usage of these terminologies is mandatory and should be corrected throughout the manuscript.

8. What was the method for detection used in the database? Was it based on polymerase chain reaction (PCR)? I would advise reading the following report and citing it among your references:

Shahriarirad R, Sarkari B. COVID-19: clinical or laboratory diagnosis? A matter of debate. Trop Doct. 2021 Jan;51(1):131-132. doi: 10.1177/0049475520945446. Epub 2020 Aug 6. PMID: 32762302.

9. The authors must add more comparisons regarding the prevalence and presentation of symptoms in their Discussion section, especially with neighboring countries and particularly during that period of the pandemic:

Shahriarirad R, Khodamoradi Z, Erfani A, Hosseinpour H, Ranjbar K, Emami Y, Mirahmadizadeh A, Lotfi M, Shirazi Yeganeh B, Dorrani Nejad A, Hemmati A, Ebrahimi M, Moghadami M. Epidemiological and clinical features of 2019 novel coronavirus diseases (COVID-19) in the South of Iran. BMC Infect Dis. 2020 Jun 18;20(1):427. doi: 10.1186/s12879-020-05128-x. PMID: 32552751; PMCID: PMC7301075.

Alasia D, Owhonda G, Maduka O, Nwadiuto I, Arugu G, Tobin-West C, Azi E, Oris-Onyiri V, Urang IJ, Abikor V, Olofinuka AM, Adebiyi O, Somiari A, Avundaa H, Alali A. Clinical and epidemiological characteristics of 646 hospitalised SARS-Cov-2 positive patients in Rivers State Nigeria: a prospective observational study. Pan Afr Med J. 2021 Jan 12;38:25. doi: 10.11604/pamj.2021.38.25.26755. PMID: 33777293; PMCID: PMC7955600

10. They should also compare their study with studies on health care workers worldwide such as:

Sabetian G, Moghadami M, Hashemizadeh Fard Haghighi L, Shahriarirad R, Fallahi MJ, Asmarian N, Moeini YS. COVID-19 infection among healthcare workers: a cross-sectional study in southwest Iran. Virol J. 2021 Mar 17;18(1):58. doi: 10.1186/s12985-021-01532-0. PMID: 33731169; PMCID: PMC7968574 

Please review and cite the mentioned references appropriately.

## Round 2 Review

The authors [1] have done a fine job in addressing their shortcomings and my previous comments. I only have a few more minor comments that need to be addressed:

1. Introduction, first paragraph: Please clarify and update what you mean by “to date.”

2. Add features of the first table to the second table and perform the related analysis. In other words, was there a significant difference in the antibody-positive and negative groups regarding working hours, gender, age, etc?

3. Just for consideration, if possible, the authors could also provide a receiver operating characteristic (ROC) curve analysis based on the hours of a shift to provide a cut-off. Alternatively, if possible, they should perform a multiple regression analysis for reporting risk factors in their study.

## Abbreviations

PCR: polymerase chain reaction
ROC: receiver operating characteristic
